# Editorial: Bio-based compounds from plants and beneficial microbes for alleviation of biotic and abiotic stress

**DOI:** 10.3389/fpls.2024.1382304

**Published:** 2024-04-10

**Authors:** Antonios Chrysargyris, Nikolaos Tzortzakis, Aziz Aziz

**Affiliations:** ^1^ Department of Agricultural Sciences, Biotechnology and Food Science, Cyprus University of Technology, Lemesos, Cyprus; ^2^ University of Reims Champagne-Ardenne, INRAE, RIBP USC 1488, Reims, France

**Keywords:** biotic and abiotic stress, microbial and plant derived compounds, natural compounds, plant pathogens, stress tolerance

## Introduction

The world’s concerning rate of population expansion, along with the increasing climatic change, has raised serious concerns about the need to ensure healthy and sustainable food production. This production is often hampered by a number of environmental constraints, including bio- and abiotic stresses, which result in a substantial decrease of crop yield and quality. In recent years, growing demand for new safer and greener solutions has received much attention in crop protection and resilience against these stresses. Harnessing bio-based compounds is now considered as a valuable tool for sustainable agriculture, thanks to their diversity and effectiveness in promoting plant growth and health (Asif et al., 2023; Priya et al., 2023).

Bio-based compounds refer to a range of different products, including those derived from plants, algae or microorganisms. They have emerged as promising solutions for enhancing agricultural sustainability and global food security thanks to their biological activities as biostimulants or biocontrol agents against the detrimental effects of both biotic and abiotic stressors (Chaudhary et al., 2022; Ng et al., 2024). However, knowledge about their mechanisms of action before or after stress challenge are still poorly understood.

The present Research Topic gathers recent studies and covers current status and future research directions regarding the use and mechanisms of action of various natural/bio-based products, including biological extracts, and compounds derived from plants and beneficial microbes, as biostimulants toward abiotic stresses or biocontrol agents of plant diseases.

## Bio-based compounds: applications and mechanisms towards biotic and abiotic stress

Plant are frequently challenged by a variety of biotic (pathogens, herbivores, etc.) and abiotic stresses (drought, high and low temperatures, heavy metals, etc.) and display adaptive responses accordingly. Salinity is a major abiotic stress that affects various physiological, biochemical, and molecular mechanisms. The research conducted by Zhou et al., on potato (*Solanum tuberosum* L.) illustrated adaptive resistance mechanisms highlighting the role of Brassinosteroids (BRs) in salt stress tolerance at the transcriptional, hormonal, physiological and phenotypical levels. Based on RNA-sequencing, the authors showed that most of the differentially expressed genes were related to the regulation of BRs metabolism, pigment metabolism, and plant hormone signal transduction. These findings suggested that the overexpression of the constitutive photomorphogenesis and dwarf (*CPD*) gene, which encodes a C-3 oxidase involved in BRs biosynthesis, can alleviate the damage caused by salt stress and enhance the saline resistance of potato plantlets.

Heavy metals including cadmium (Cd) raise major concerns for agriculture, human health and environment. Developing effective strategies to lessen the harmful impacts of Cd remains one of the main challenges. In this context, Haider et al. investigated the combined effects of biochar and beneficial microorganisms (*Trichoderma harzianum*-fungus and *Bacillus subtilis*-bacteria), on stabilization of Cd and maize growth in contaminated soil. Authors reported that the combination of biochar and microorganism was more effective on plant growth promotion, water use efficiency and photosynthetic rates, compared to single treatment against Cd stress and toxicity. However, such application might have limitations for the biochar effectiveness, due to the aging factor and biochar leaching alkalinity. Further experiments are needed to understand biochar and microbial efficiency for specific bioremediation.

Plants can activate various defense mechanisms by producing different metabolites to cope with pathogens and pests. Aguiar et al. explored the chemical diversity of bioactive compounds in soybean (*Glycine max*) cultivars against *Spodoptera cosmioides* caterpillars. It was reported that resistant cultivars to *S. cosmioides* accumulated molecules related to the jasmonic and salicylic acid pathways. Overall, the main soybean defense strategy may involve a higher cell membrane lipid biosynthesis, storage of molecules in inactive form, and several fatty acid products that serve as defense signals, including green leaf volatiles. This provided evidence that the biosynthesis of lipids and isoflavones in *G. max* may directly be related to the constitutive resistance against *S. cosmioides* caterpillars. Therefore, primed plants display a faster and more robust activation of defense response, thereby hindering the herbivory attacks.

The bio-based products are also used as biostimulants to counteract the detrimental effects of abiotic stress. However, the underlying molecular mechanisms remain unclear. In that sense, Chambard et al. compared the effect of living *Saccharomyces cerevisiae*-based biostimulant, as seed coating in two different soybean cultures, using transcriptomic analysis. It was observed that the living yeast-based biostimulant triggered similar pathways, with upregulation of common genes in the two cultures. Overall, the activated pathways are related to abiotic stress tolerance and cell wall/carbohydrate synthesis, maintaining higher levels of sugars. Moreover, a review by Talavera-Mateo et al. proposed insights for the seed defense priming as an efficient and reliable approach for pathogen protection and pest management. The authors highlighted that biological isolates and naturally occurring plant substances should have a greater impact on plant and antagonist effectiveness than synthetic chemicals and volatiles. Seed defense priming does not seem to undergo the initial penalty in fitness due to resource allocation that occurs with primed seedlings or adult plants. They also reported that seed defense priming is likely to grant plants a more powerful resistance against fungi than against bacteria and herbivores, as well as to affect more positively to cereals and crops than woody and herbaceous plants.

Another aspect dealing with the effectiveness of natural based products as a biocontrol agent against pathogenic oomycetes has also been addressed by Mian et al. The authors reported that leaf extracts from seven *Eruca vesicaria* subsp. *sativa* cultivars, as well as their biochemically active compounds (glucosinolates and downstream-derived products) inhibited mycelial growth of pathogenic oomycetes (*Phytopythium chamaehyphon*, *Phytopythium vexans* and *Phytophthora citrophthora*) causing diseases in kiwifruit. Even though *in vitro* tests are typically just the beginning of a lengthy process, this work serves as a starting point for more field research.

## Concluding remarks and future perspective

The current Research Topic offers significant advances on the function of bio-based products in promoting plant growth and health and emphasizes the intricate interactions among various factors that influence plant adaptive and defense mechanisms to cope with biotic and abiotic stress. The compiled information revealed a growing interest for bio-based solutions, including biostimulants and microbe-based inoculants, as promising tools to increase plant resilience/resistance, thereby contributing to a more sustainable crop production in a changing environment. The importance of bio-based-triggered priming plants for enhanced signaling pathways, defense responses and metabolite accumulation has been widely emphasized as a beneficial and innovative approach, combining efficiency and energy cost reduction. However, molecular and physiological mechanisms involved in the priming state remain to be elucidated. Such mechanisms implies that the application of bio-based compounds could have a beneficial potential for agriculture and environmental remediation. Further research is also required to elucidate the implication of bio-based products in priming state after stress challenge, and especially the impact of bio-based products on local rhizospheric microbial communities.

## Author contributions

AC: Conceptualization, Visualization, Writing – original draft, Writing – review & editing. NT: Conceptualization, Visualization, Writing – original draft, Writing – review & editing. AA: Conceptualization, Visualization, Writing – original draft, Writing – review & editing.
